# Combined Developmental Toxicity of Ecologically Relevant Concentrations of the PFOS Alternative F-53B and Hexavalent Chromium in Zebrafish, Danio rerio

**DOI:** 10.3390/toxics14060471

**Published:** 2026-05-27

**Authors:** Qunjie Feng, Ximei Wu, Ming Chen, Hui Li, Wei Tong, Yanhong Gao, Wenli Li, Zenghua Qi, Chaoyang Long, Yingxin Yu

**Affiliations:** 1 Guangdong-Hong Kong-Macao Joint Laboratory for Contaminants Exposure and Health, Guangdong Provincial Center for Disease Control and Prevention, Guangzhou 510430, China; 2 Guangdong-Hong Kong-Macao Joint Laboratory for Contaminants Exposure and Health, Guangdong Key Laboratory of Environmental Catalysis and Health Risk Control, Institute of Environmental Health and Pollution Control, Guangdong University of Technology, Guangzhou 510006, China; 3 Guangdong Basic Research Center of Excellence for Ecological Security and Green Development, Key Laboratory of City Cluster Environmental Safety and Green Development, School of Environmental Science and Engineering, Guangdong University of Technology, Guangzhou 510006, China

**Keywords:** co-exposure, ocular development, retinal histology, locomotor behavior, retinoic acid signaling, emerging contaminants

## Abstract

6:2 Chlorinated polyfluoroether sulfonate (F-53B), an emerging per- and polyfluoroalkyl substance used as a perfluorooctane sulfonate substitute in electroplating, may co-occur with Cr(VI) in industrially affected aquatic environments. However, their combined developmental toxicity in vertebrates remains poorly understood. This study compared the effects of F-53B and Cr(VI) alone and in combination on zebrafish embryos and larvae exposed from 0 to 120 hpf at environmentally relevant concentrations (10 and 100 μg/L). Developmental toxicity, ocular morphology, retinal histology, locomotor behavior, and ocular-development-related gene expression were assessed. Single Cr(VI) exposure produced prominent effects on hatching, locomotor activity, retinal-layer thickness, and selected transcriptional responses related to retinoic acid signaling and ocular development. F-53B alone caused developmental and ocular alterations but generally produced weaker or more selective responses than Cr(VI). Co-exposure did not uniformly intensify the toxicity of either compound. Instead, the mixture enhanced some endpoints, including malformation, heart rate, and specific ocular or retinal alterations while showing weaker or divergent responses for other endpoints, such as locomotor activity and selected transcriptional markers. These findings indicate that F-53B/Cr(VI) co-exposure produces endpoint-dependent toxicity rather than a consistent synergistic pattern. Overall, the study highlights the importance of comparing single and combined exposures across multiple biological endpoints when assessing the developmental risks of co-occurring contaminants.

## 1. Introduction

Hexavalent chromium (Cr(VI)) is extensively used in electroplating and other metal-finishing processes, and has consequently become a common contaminant in industrial wastewater and aquatic environments impacted by these activities [1]. Because electroplating operations commonly employ fluorinated chromium-mist suppressants alongside chromium-based processes, wastewater and receiving waters are susceptible to simultaneous contamination by Cr(VI) and PFAS-derived surfactants or their substitutes, creating a realistic co-occurrence scenario in industrially impacted aquatic environments [2,3]. In addition, monitoring data indicate that environmentally relevant chromium burdens persist in surface waters, with regional surveys reporting chromium levels of 22–185 μg/L in certain areas of China [4]. The U.S. Environmental Protection Agency (EPA) recommends dissolved Cr(VI) aquatic-life criteria of 16 µg/L for acute freshwater exposure and 11 µg/L for chronic freshwater exposure, with corresponding saltwater criteria of 1100 and 50 µg/L, respectively [5]. The Canadian Council of Ministers of the Environment (CCME) has established more stringent long-term guidelines for Cr(VI), with values of 1.0 µg/L for freshwater life and 1.5 µg/L for marine life [6]. Since PFOS was listed as a persistent organic pollutant under the Stockholm Convention in 2009, regulatory restrictions on PFOS have accelerated the search for alternative chromium-mist suppressants in the electroplating industry [7]. One prominent substitute is 6:2 chlorinated polyfluoroether sulfonate (6:2 Cl-PFESA, F-53B), which is widely used in China as a chromium-mist suppressant to reduce occupational exposure during electroplating operations [8,9]. Consistent with this use pattern, F-53B has been repeatedly detected in riverine waters (1.9–40.2 ng/L) and electroplating effluents (43–112 μg/L) [9,10]. Taken together, these field and effluent data suggest that aquatic organisms in electroplating-impacted systems may face a substantial risk of co-exposure to Cr(VI) and F-53B.

Single-chemical evidence indicates that both F-53B and Cr(VI) can perturb early development and neurobehavior in fish. For F-53B, reported adverse outcomes include embryotoxicity, reproductive toxicity, and the disruption of thyroid hormone homeostasis [8,10,11]. In zebrafish embryos exposed to F-53B at ng/L levels, abnormal retinal layering and related swimming deficits have been observed, accompanied by the downregulation of genes involved in retinoic acid (RA) signaling, lens formation, and retinal development, which is consistent with interference with RA-dependent visual-pathway maturation [12]. Beyond ocular endpoints, F-53B exposure could impair neurodevelopment and suppress locomotor activity during critical developmental windows, with evidence linking hypoactivity to dopaminergic-system injury [13]. Cr(VI) exposure, meanwhile, has been shown to impair somitogenesis and organogenesis in zebrafish embryos, producing severe malformations and acute lethality at higher concentrations [14,15]. Exposed embryos and hatched larvae could exhibit growth retardation and altered locomotor performance, including anxiety-like behavioral phenotypes [14,15]. However, evidence from single-compound exposure studies is insufficient to predict whether combined exposure will produce additive, antagonistic, or synergistic effects in sensitive developmental pathways.

In contrast, the joint toxic effects and underlying mechanisms of F-53B and Cr(VI) co-exposure in higher aquatic organisms have not yet been clearly elucidated. Evidence from mixture contexts suggests that co-occurring pollutants could produce non-additive outcomes through changes in bioavailability and redox homeostasis. For example, polystyrene microplastics can adsorb F-53B and heavy metals, modulating their bioaccumulation and enhancing inflammatory and oxidative stress responses in aquatic organisms [16,17,18]. In addition, low-dose combinations of Cd and imidacloprid during early development elevate retinal apoptosis and induce antioxidant-defense genes, consistent with mixture-driven oxidative stress and impaired retinal development [19]. In a microbial model, concurrent F-53B and Cr(VI) contamination damages *Pseudomonas aeruginosa* biofilms more severely than either pollutant alone, compromising membranes and matrix and driving excessive intracellular ROS with compensatory antioxidant-enzyme responses [20]. Collectively, these findings imply that mixtures containing F-53B and metals might disrupt redox balance and developmental processes in a non-additive manner. However, the consequences of F-53B/Cr(VI) co-exposure for vertebrate visual-system development and function have not yet been systematically investigated.

Therefore, in the present study, F-53B and Cr(VI) were selected as representative co-occurring contaminants in electroplating-impacted waters to investigate their combined developmental and visual toxicity in zebrafish under ecologically relevant exposure conditions. The effects of single and combined exposures on developmental toxicity, locomotor behavior, ocular morphology, retinal histopathology, and transcriptional responses of RA signaling- and vision-related genes were compared. This study was designed to determine whether co-exposure induces endpoint-dependent interactions and to provide experimental evidence relevant to the ecological risk assessment of realistic contaminant mixtures in electroplating-associated aquatic environments.

## 2. Materials and Methods

### 2.1. Reagents and Materials

F-53B (≥98%) was obtained from Shanghai Maikun Chemical Co., Ltd. (Shanghai, China), and Cr(VI) was supplied as potassium dichromate (K_2_Cr_2_O_7_, analytical grade, ≥98%; Macklin Biochemical Technology Co., Ltd., Shanghai, China). Dimethyl sulfoxide (DMSO, ≥99.9%) and the anesthetic MS-222 (≥98%) were purchased from Sigma-Aldrich (St. Louis, MO, USA). F-53B was dissolved in DMSO as a co-solvent. A concentrated F-53B stock solution (1000 mg/L) was prepared in DMSO, stored in the dark at 4 °C, and diluted in standard reconstituted water (SRW) on the day of use to obtain working solutions. The K_2_Cr_2_O_7_ stock solution was prepared directly in SRW and similarly diluted immediately before use. All exposure solutions were freshly prepared and completely renewed every 24 h to minimize concentration drift due to uptake or degradation.

### 2.2. Zebrafish Husbandry and Embryo Collection

Sexually mature zebrafish (*Danio rerio*, AB strain; 6–12 months old) were maintained in a recirculating, filtered system at a density of 10–15 fish per 3 L tank, under the following conditions: 28 ± 1 °C, pH 6.5–7.5, conductivity 150–550 μS/cm, and a 14 h light/10 h dark photoperiod. Fish were fed *Artemia nauplii* twice daily. The adult fish system was supplied with dechlorinated and filtered freshwater. For embryo rinsing, incubation, and exposure, SRW was freshly prepared using ultrapure water and analytical-grade salts. The SRW contained 63.0 mg/L NaHCO_3_, 5.5 mg/L KCl, 294.0 mg/L CaCl_2_·2H_2_O, and 123.3 mg/L MgSO_4_·7H_2_O. The pH was adjusted to 7.2–7.4 before use.

For spawning, females and males were placed in breeding tanks at a ratio of 1:2 and separated by a vertical divider at 17:00 on the day before mating, with the water level maintained below half the divider height. At 08:00 the following morning, the divider was removed and lights were turned on to induce spawning. Fertilized eggs were collected within 0.5 h, while unfertilized and dead eggs were removed. Embryos were gently rinsed with SRW to remove debris, and 2 h post-fertilization (hpf) was designated as the start of the exposure experiment. Cleaned embryos were transferred to pre-warmed (28 °C) SRW and incubated in a temperature-controlled chamber.

### 2.3. Exposure Protocols

For the main developmental toxicity experiment, exposure levels were selected based on environmentally relevant concentrations of F-53B and Cr(VI) and the results of preliminary tests [4,9,10]. Exposure groups were established as follows: untreated control (SRW only), vehicle control (VC; SRW containing 0.01% DMSO), single-compound exposures FB-L (F-53B, 10 μg/L), FB-H (F-53B, 100 μg/L), Cr-L (Cr(VI), 10 μg/L), and Cr-H (Cr(VI), 100 μg/L), and matched co-exposures Mix-L (F-53B + Cr(VI), 10 + 10 μg/L) and Mix-H (F-53B + Cr(VI), 100 + 100 μg/L). In the group names, FB denotes F-53B, Cr denotes Cr(VI), and Mix denotes matched F-53B/Cr(VI) co-exposure; L and H indicate the low-dose (10 μg/L) and high-dose (100 μg/L) exposure levels, respectively. All exposure solutions and VC were adjusted to the same final DMSO concentration (0.01%). Because all treated groups contained 0.01% DMSO, VC was used as the primary comparator for treatment effects. Comparisons between the untreated control and VC are provided in Table S1.

Zebrafish embryos/larvae were continuously exposed for 120 h in the corresponding SRW-based exposure solutions. For each group, 200 healthy embryos at 2 hpf were used in each of three independent biological replicates and exposed until 120 hpf. Developmental endpoints were assessed in 6-well plates, with ten embryos placed in each well containing 5 mL of exposure solution. The embryo density was controlled to avoid crowding and hypoxia. All exposure media, including F-53B, Cr(VI), mixture solutions, VC, and control media, were completely renewed every 24 h. All exposures were conducted at 28 ± 1 °C and the pH of the SRW-based exposure media was maintained at 7.2–7.4, and dead embryos were removed promptly to ensure data quality. Concentrations of F-53B and Cr(VI) in the exposure media were quantified during the experiment (Table S2). Detailed analytical procedures are provided in the Supplementary Materials (Text S1).

### 2.4. Developmental Toxicity

Embryos were monitored until 120 hpf, and survival, hatching, and malformations were recorded over time, with quantitative assessments at key time points. Hatching was evaluated primarily at 72 hpf, supplemented by daily observations. Unfertilized and dead eggs were excluded from the denominators so that rates reflected only individuals alive at each assessment. Death was defined as embryo coagulation or cessation of heartbeat confirmed by continuous microscopic observation. Malformations were scored based on visible criteria, including pericardial/yolk-sac edema, axial and tail curvature, and localized hemorrhage or generalized edema. Malformation rate was calculated as the number of malformed larvae divided by the number of surviving larvae. All observations were conducted at 28 ± 1 °C under uniform illumination and pH 6.5–7.5 and were completed before daily renewal of the exposure medium to minimize positional or microenvironmental bias.

To further evaluate growth and functional development, larvae were lightly anesthetized with MS-222 at 120 hpf, imaged under a calibrated microscope, and analyzed in ImageJ (version 1.53c; National Institutes of Health, Bethesda, MD, USA) for body length. At 24 hpf, spontaneous tail coiling was recorded as the number of tail flicks per embryo over a 1-minute period. At 72 hpf, heart rate was quantified as beats per minute.

### 2.5. Ocular Morphometry and Retinal Histology

At 120 hpf, twenty larvae per group were collected for external morphometric analysis, fixed in 10% neutral buffered formalin for 24 h, and imaged under a light microscope. Body and ocular morphometrics were quantified in ImageJ, including body length, head width, and horizontal and vertical eye diameters. The measurements of head width and horizontal and vertical eye diameters are illustrated in Figure S1A [21]. Body length was defined as the distance from the anterior edge of the head to the tip of the tail.

For retinal histological assessment, twelve larvae from each replicate were randomly selected. After euthanasia, eyes were dissected, fixed in 10% neutral buffered formalin for 24 h, dehydrated through a graded ethanol series, and embedded in paraffin. Serial sections (4 μm) were prepared, stained with hematoxylin and eosin (H&E) (Servicebio, Wuhan, China), and then examined under a light microscope. Retinal layer thicknesses, including the ganglion cell layer (GCL), inner plexiform layer (IPL), inner nuclear layer (INL), and outer nuclear layer (ONL), together with total retinal thickness (GCL + IPL + INL + ONL), were measured using ImageJ. Lens thickness was measured separately, and the corresponding structures are shown in Figure S1B.

### 2.6. Larval Light–Dark Assay

At 120 hpf, larvae with normal morphology, spontaneous motility, and no obvious malformations were subjected to a standardized light–dark–light locomotor assay using a ViewPoint zebrafish tracking system (ViewPoint, Lyon, France). Both minute-by-minute and phase-averaged swimming speeds were recorded. Prior to testing, individual larvae were transferred to 24-well plates, with one larva per well, and acclimated for 30 min under laboratory conditions. The stimulus sequence consisted of 0–10 min light (Light_1_), 10–20 min dark (Dark), and 20–30 min light (Light_2_). Temperature was maintained at 28 ± 1 °C, and mechanical and visual disturbances were minimized. For each treatment group, 24 larvae were analyzed using the same camera and detection parameters. Raw position data were collected at 1-minute intervals and used to calculate swimming speed.

### 2.7. Gene Expression Analysis

Gene expression analysis focused on functional pathways associated with ocular development and visual toxicity, including RA signaling, lens differentiation, retinal development, and phototransduction. Procedures for total RNA isolation, reverse transcription, and quantitative real-time PCR (qPCR) are described in detail in the Supplementary Materials (Text S2). *β-Actin* served as the internal reference gene.

The qPCR panel included genes related to RA metabolism and signaling, lens development, retinal development, and phototransduction. The gene symbols, full names, and functional categories of all target and reference genes are summarized in Table 1. Primer sequences for these genes are provided in Table S3.

### 2.8. Statistical Analysis

Statistical analyses were performed using IBM SPSS Statistics 20.0 (IBM Corp., Armonk, NY, USA). Data are presented as mean ± standard deviation (SD), and *p* < 0.05 was considered statistically significant. Normality and homogeneity of variance were assessed using the Shapiro–Wilk test and Levene’s test, respectively. When necessary, square-root or reciprocal transformation was applied before final analysis.

The untreated control was used to evaluate potential vehicle-related effects, whereas the main exposure analysis used the vehicle control (VC) as the primary comparator because all exposure groups contained 0.01% DMSO. Depending on data distribution and variance homogeneity, comparisons were performed using the Student’s *t*-test, Welch’s *t*-test, the Mann–Whitney U test, one-way ANOVA, Welch’s ANOVA, or the Kruskal–Wallis test, followed by appropriate post hoc tests. Effect sizes were reported as η^2^ for ANOVA-family tests and ε^2^ for Kruskal–Wallis tests. Detailed assumption checks, transformation decisions, statistical tests, post hoc results, *p* values, and effect sizes are provided in Table S4 (Supplementary Materials, SM2, Excel format). Sample size and replicate number were determined with reference to previous zebrafish developmental toxicity studies using comparable endpoints [8,12]. A sensitivity analysis was further performed using G*Power software (version 3.1.9.7; Heinrich Heine University Düsseldorf, Düsseldorf, Germany) to evaluate the detectable effect sizes of the present design. The minimum detectable effect size ranged from approximately *f* = 0.341 to *f* = 0.995, depending on the endpoint-specific sample size. Detailed results are provided in Table S5 (SM2, Excel format).

## 3. Results

### 3.1. Effects of F-53B and Cr(VI) Alone or in Combination on Developmental Toxicity in Zebrafish

No statistically significant differences were observed between the untreated control and VC for the analyzed endpoints (Table S1). Because all exposure solutions were adjusted to 0.01% DMSO, VC was used as the primary comparator for the treatment effects described below.

Across developmental endpoints, single exposure and co-exposure produced distinct toxicity profiles. Cr(VI) generally produced stronger developmental disturbance than F-53B at matched concentrations, whereas mixture exposure did not uniformly increase all developmental endpoints. Instead, the mixture was most clearly associated with enhanced malformation and increased heart rate, while mortality and early motor activity did not show consistent potentiation under co-exposure (Table 2).

**Figure 1:**
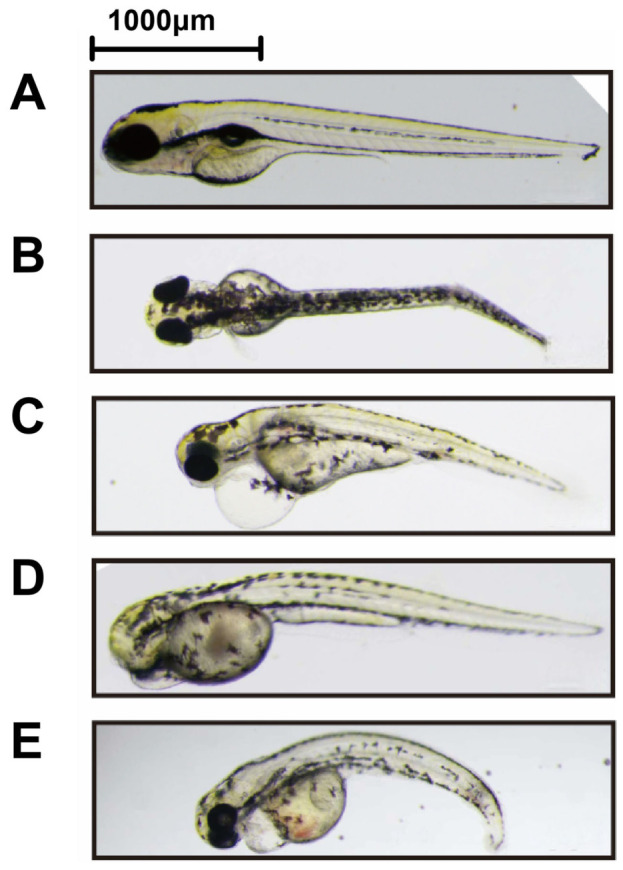
Representative malformations observed in zebrafish larvae after 120-h exposure to F-53B, Cr(VI), or their mixture. (**A**) Normal larva; (**B**) spinal curvature (SC); (**C**) pericardial edema (PE); (**D**) anophthalmia with yolk-sac edema (YSE); (**E**) severe SC with hemorrhage and generalized edema.

Hatching success was significantly reduced relative to VC in the FB-L, Cr-L, Mix-L, Cr-H, and Mix-H groups, with the strongest difference observed in the Mix-L group (*p* < 0.001). However, the hatching response did not follow a simple concentration-dependent pattern across either single-exposure or mixture-exposure groups. In contrast, malformation rate was significantly increased in all exposure groups relative to VC, with FB-L showing significance at *p* < 0.01 and the remaining exposure groups showing *p* < 0.001. Among the treatment groups, Mix-H showed a higher malformation rate than FB-L, Cr-L, Mix-L, and FB-H, whereas Mix-L differed from Cr-H and Mix-H. Representative deformities observed in the present study included axial curvature, pericardial edema, anophthalmia with yolk-sac edema, and severe axial curvature with hemorrhage and generalized edema (Figure 1A–E).

Cumulative mortality was significantly increased relative to VC in all exposure groups (*p* < 0.001). However, unlike malformation, the mixture groups did not consistently exceed the corresponding single-exposure groups. Early functional endpoints showed endpoint-specific responses to co-exposure. Spontaneous coiling did not differ significantly from VC in any exposure group, and Cr-L and Cr-H showed significantly lower coiling frequencies than Mix-L (*p* < 0.05), indicating that co-exposure did not further suppress early motor activity compared with Cr(VI) alone (Table 2). In contrast, heart rate at 72 hpf was increased most markedly in the mixture groups. Heart rate was significantly increased relative to VC in the Cr-L, Mix-L, FB-H, Cr-H, and Mix-H groups, with Mix-L and Mix-H showing the strongest differences (*p* < 0.001). Mix-L also differed from FB-L (*p* < 0.01), and Mix-H differed from FB-L (*p* < 0.01) and FB-H (*p* < 0.05). Body length showed only a limited response, with a significant increase observed only in Mix-H relative to VC (*p* < 0.05).

### 3.2. Effects of F-53B and Cr(VI) Alone or in Combination on Ocular Morphometrics and Retinal Histomorphology in Zebrafish Larvae

At 5 dpf, ocular morphometric and retinal histological endpoints also revealed clear differences between single exposure and co-exposure. External ocular enlargement was observed in several exposure groups, but the affected eye-diameter axis and treatment pattern differed among F-53B, Cr(VI), and the mixture (Table 2). For the *X*-axis eye diameter, significant increases relative to VC were observed in FB-L and in all 100 μg/L exposure groups, including FB-H, Cr-H, and Mix-H (all *p* < 0.01). For the *Y*-axis eye diameter, significant increases were observed only in FB-L (*p* < 0.05) and Mix-H (*p* < 0.001). Thus, F-53B alone produced a low-dose ocular enlargement response, whereas high-dose co-exposure produced the most evident increase in *Y*-axis eye diameter.

**Figure 2:**
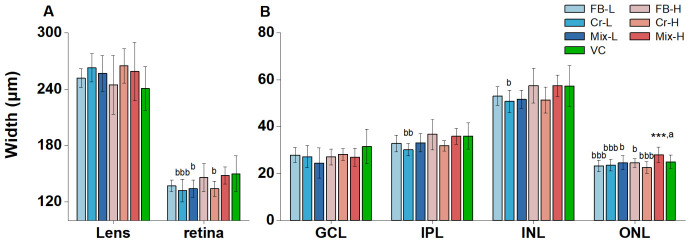
Quantitative measurements of lens and retinal layer thickness in 5 dpf zebrafish larvae after 120-h exposure to F-53B, Cr(VI), or their mixture. (**A**) Lens thickness and total retinal thickness. (**B**) Thicknesses of retinal layers, including ganglion cell layer (GCL), inner plexiform layer (IPL), inner nuclear layer (INL), and outer nuclear layer (ONL). Each treatment group contained 12 larvae. Statistical significance is indicated as follows: *** *p* < 0.001 compared with VC; ^a^ *p* < 0.05 compared with Mix-L; and ^b^ *p* < 0.05, ^bb^ *p* < 0.01, ^bbb^ *p* < 0.001 compared with Mix-H.

Histological analysis further showed that the external ocular enlargement was accompanied by group-specific retinal alterations (Figure 2). No significant difference in lens thickness was detected among the analyzed group comparisons. Although total retinal thickness did not differ significantly from VC, Cr-L and Cr-H showed significantly lower total retina thickness than Mix-H (*p* < 0.001 and *p* < 0.05, respectively), and Mix-L was also thinner than Mix-H (*p* < 0.05). These comparisons indicate that Cr(VI)-only exposure was more closely associated with retinal thinning than high-dose co-exposure.

Layer-specific analysis supported this distinction. For IPL and INL thickness, Cr-L showed significantly reduced thickness relative to Mix-H (*p* < 0.01 and *p* < 0.05, respectively). Therefore, high-dose co-exposure did not aggravate Cr(VI)-associated thinning of these retinal layers (Figure 2B). Instead, Mix-H showed a different retinal pattern, characterized by significant ONL thickening relative to VC (*p* < 0.001). Mix-H also differed from Mix-L (*p* < 0.05), FB-H (*p* < 0.05), FB-L (*p* < 0.001), Cr-L (*p* < 0.001), and Cr-H (*p* < 0.001). These results indicate that co-exposure did not simply intensify the retinal effects of Cr(VI). Rather, the mixture shifted the retinal response from Cr(VI)-associated thinning toward a distinct pattern involving ONL thickening.

### 3.3. Effects of F-53B and Cr(VI) Alone or in Combination on Larval Locomotor Activity

**Figure 3:**
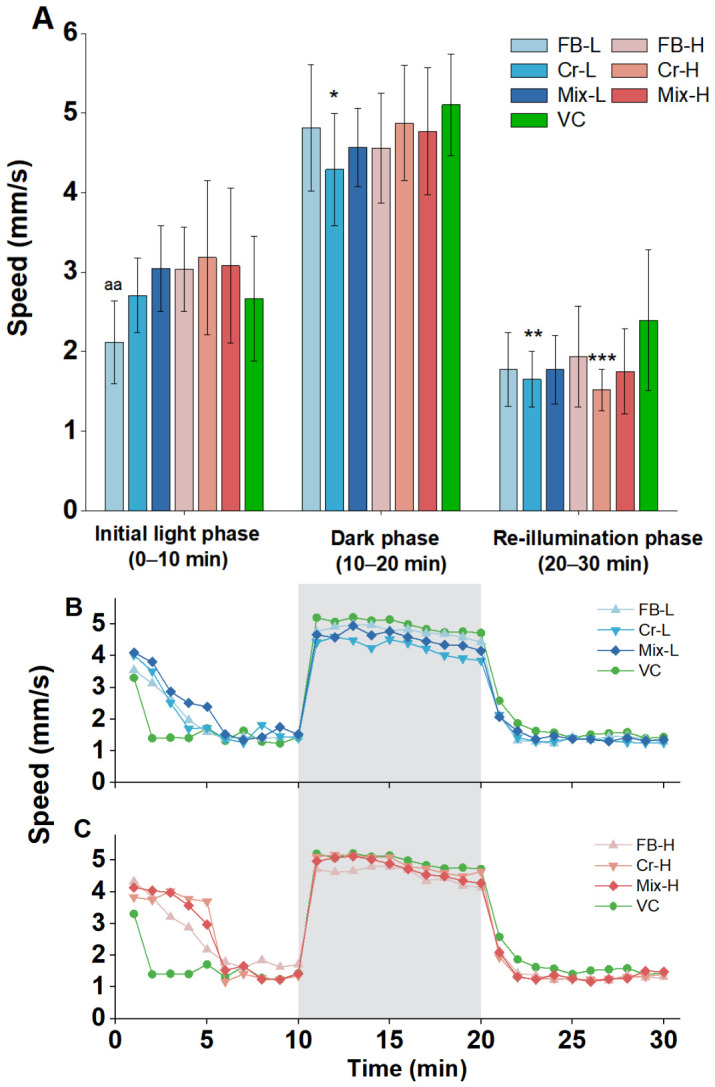
Locomotor activity of zebrafish larvae in a light–dark–light assay at 5 dpf after 120-h exposure to F-53B, Cr(VI), or their mixture. (**A**) Average swimming speed during the assay; (**B**) representative locomotor traces for VC and low-dose groups; (**C**) representative locomotor traces for VC and high-dose groups. Light_1_: 0–10 min light phase; Dark: 10–20 min dark phase; Light_2_: 20–30 min light phase; *n* = 24 per group. Statistical significance is indicated as follows: * *p* < 0.05, ** *p* < 0.01, *** *p* < 0.001 compared with VC; ^aa^ *p* < 0.01 compared with Mix-L.

In the light–dark locomotor assay, the main behavioral effect was driven by Cr(VI), and co-exposure did not produce stronger locomotor inhibition than Cr(VI) alone (Figure 3). During the first light phase, no exposure group differed significantly from VC, although FB-L differed from Mix-L (*p* < 0.01). During the dark phase, reduced swimming speed was observed only in the Cr-L group relative to VC (*p* < 0.05). During the second light phase, significant locomotor inhibition was observed in Cr-L (*p* < 0.01) and Cr-H (*p* < 0.001) relative to VC, whereas neither mixture group differed significantly from VC. These results indicate that Cr(VI) alone produced the clearest locomotor impairment, particularly during the dark phase and the second light phase. In contrast, the mixture groups did not show greater impairment than the corresponding Cr(VI)-only groups.

### 3.4. Exposure to F-53B and Cr(VI) Alone or in Combination Alters Gene Expression

**Figure 4:**
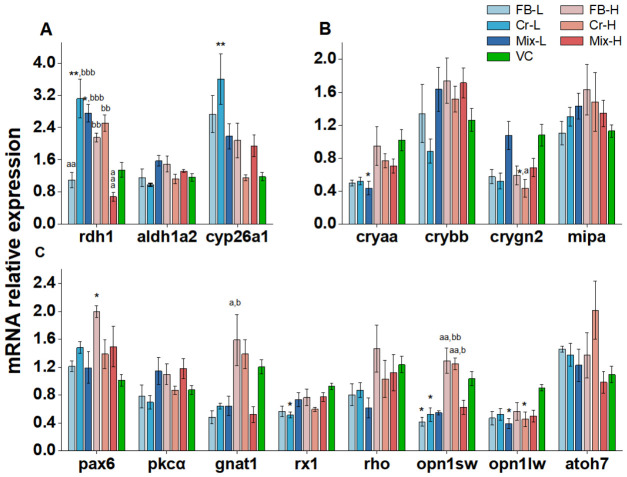
Relative mRNA expression of retinoic acid (RA) signaling, lens-development, retinal-development, and phototransduction genes in zebrafish larvae following 120-h exposure to F-53B, Cr(VI), or their mixture. (**A**) RA metabolism and signaling genes; (**B**) lens development markers; (**C**) retinal-development and phototransduction genes. Each exposure condition was tested in triplicate. Statistical significance is indicated as follows: * *p* < 0.05, ** *p* < 0.01 compared with VC; ^a^ *p* < 0.05, ^aa^ *p* < 0.01, ^aaa^ *p* < 0.001 compared with Mix-L; and ^b^ *p* < 0.05, ^bb^ *p* < 0.01, ^bbb^ *p* < 0.001 compared with Mix-H.

To further interpret the ocular and retinal alterations observed at the morphological and histological levels, transcriptional responses in RA signaling and ocular development pathways were assessed after exposure through 5 dpf (Figure 4). The qPCR results showed that single exposure and co-exposure affected overlapping but non-identical transcriptional endpoints.

Within the RA signaling pathway, Cr-L significantly increased both *cyp26a1* and *rdh1* expression relative to VC (both *p* < 0.01), while Mix-L significantly increased only *rdh1* expression relative to VC (*p* < 0.05). At 100 μg/L, *rdh1* expression in FB-H and Cr-H was higher than that in Mix-H (both *p* < 0.01), and Cr-L and Mix-L also differed from Mix-H (both *p* < 0.001), indicating attenuated *rdh1* responses in the high-dose mixture group (Figure 4A).

Lens-related transcripts also showed endpoint-specific responses. *cryaa* was significantly downregulated only in the Mix-L group relative to VC (*p* < 0.05), rather than across all 10 μg/L treatments. In contrast, *crygn2* expression was significantly lower in Cr-H than in the VC and Mix-L groups (both *p* < 0.05), suggesting a Cr(VI)-dominant response at the high dose.

Photoreceptor- and cone-related genes showed selective but non-uniform changes. *opn1sw* was significantly downregulated in FB-L and Cr-L relative to VC (both *p* < 0.05), while FB-H and Cr-H differed from Mix-L (both *p* < 0.01) and Mix-H (*p* < 0.01 and *p* < 0.05, respectively). *opn1lw* was significantly downregulated in Mix-L and Cr-H relative to VC (both *p* < 0.05). *gnat1* did not differ significantly from VC, but FB-H differed from both Mix-L and Mix-H (both *p* < 0.05). These results indicate that F-53B, Cr(VI), and the mixture affected different components of visual-system-related transcription, rather than producing a single uniform expression pattern.

For ocular-development-related genes, *rx1* was significantly downregulated only in the Cr-L group relative to VC (*p* < 0.05), and this effect was not observed in Cr-H or either mixture group. *pax6* was significantly increased only in FB-H relative to VC (*p* < 0.05). In contrast, *pkcα* and *rho* showed no significant changes relative to VC or the corresponding mixture groups (Figure 4C).

Because the qPCR endpoints included three biological replicates per group, the G*Power-based sensitivity analysis indicated that these endpoints were powered to detect only very large omnibus effects (*f* ≈ 0.995; Table S5). Therefore, non-significant transcriptional results should be interpreted as no statistically reliable change detected under the present qPCR design, rather than as definitive evidence of no response.

## 4. Discussion

The present study compared the effects of F-53B and Cr(VI) alone and in combination on zebrafish early development across organismal, ocular, retinal, behavioral, and transcriptional endpoints. Overall, co-exposure did not simply amplify the toxicity of either compound. Instead, the mixture changed the toxicity profile in an endpoint-dependent manner. F-53B alone produced measurable but relatively selective effects, particularly on developmental and ocular morphometric endpoints and selected visual-system-related transcripts. Cr(VI) alone showed more prominent effects on hatching suppression, locomotor inhibition, retinal-layer thinning, and several RA- and ocular-development-related transcriptional responses. In contrast, F-53B/Cr(VI) co-exposure was more clearly associated with enhanced malformation, increased heart rate, and specific retinal or ocular alterations, while some Cr(VI)-dominant behavioral, retinal, and transcriptional responses were not further aggravated. These comparisons demonstrate that combined-exposure assessment provides information beyond single-compound testing by revealing endpoint shifts, attenuation of selected single-compound responses, and distinct toxicity patterns that would not be predicted from either compound alone.

For developmental toxicity, F-53B, Cr(VI), and their mixtures all produced measurable adverse developmental effects, but the single-exposure and co-exposure groups differed in the dominant endpoints affected. The observed deformities, including axial curvature, pericardial edema, hemorrhage or generalized edema, and occasional ocular defects, are consistent with previous reports for F-53B [8,22] and Cr(VI) [23]. Hatching impairment was observed in several exposure groups, but it did not show a simple monotonic concentration–response pattern. This differs from the clearer adverse trends observed for mortality and malformation. Such a pattern is biologically plausible because hatching is a time-dependent developmental process regulated by chorion structure, hatching-enzyme activity, embryonic movement, and developmental timing. Cr(VI) has been associated with reduced hatching success through the inhibition of hatching enzymes or chorion damage [23], whereas F-53B has been reported to delay chorion rupture at environmentally relevant concentrations, possibly through thyroid hormone dysregulation [24,25]. The chorion also acts as a protective barrier that permits water and gas exchange while restricting the entry of many xenobiotics, and chemicals with different physicochemical properties may interact with or penetrate the chorion to different extents [26]. In addition, toxicants can delay hatching without necessarily producing proportional increases in mortality or malformation [27]. Consistent with this complexity, the OECD Fish Embryo Test guideline treats hatching as an interpretive endpoint rather than an endpoint for LC_50_ calculation [28]. Therefore, the hatching results are best interpreted as evidence of developmental delay or chorion-related disturbance, rather than as a direct concentration-dependent ranking of overall toxicity.

Mortality and malformation further illustrate the difference between single exposure and co-exposure. Malformation was increased in all exposure groups, and the high-dose mixture showed the most pronounced malformation response among the reported pairwise comparisons. This suggests that combined exposure increased the likelihood of visible developmental abnormalities at the whole-organism level. In contrast, cumulative mortality was elevated across exposure groups, but the mixtures did not consistently exceed the corresponding single-compound exposures. Thus, the mixture enhanced some developmental outcomes, such as malformation, but did not produce a uniform increase in lethality. This distinction is important because it shows that F-53B/Cr(VI) co-exposure cannot be described by a simple toxicity hierarchy in which mixture exposure is always more severe than single exposure. The absence of a uniform synergistic pattern is also consistent with the current understanding of mixture toxicity. Recent zebrafish mixture studies have shown that combined exposures may produce synergistic, additive, or antagonistic outcomes depending on dose ratio, endpoint, developmental stage, and toxicological mode of action [29,30]. For example, Hu et al. reported antagonistic responses in several cadmium–pesticide mixtures in zebrafish larvae and suggested that differences in mode of action and mixture-induced changes in uptake or detoxification may contribute to non-additive effects [29]. In the present study, the apparent less-than-additive or antagonism-like responses observed for selected endpoints may reflect endpoint-specific response ceilings, different developmental timing, selective loss of sensitive embryos before later endpoint assessment, or toxicokinetic interactions. Overall, the developmental data indicate that co-exposure shifted the pattern of toxicity rather than uniformly increasing all adverse outcomes.

The ocular and retinal findings also demonstrate that mixture exposure produced a toxicity pattern distinct from either single compound alone. External eye enlargement was observed in several exposure groups, with the *X*-axis eye diameter increased in FB-L and all high-dose exposure groups, whereas the *Y*-axis eye diameter increased mainly in FB-L and Mix-H. These findings suggest that F-53B and co-exposure contributed to external ocular enlargement, but the response was not simply dose-dependent across all treatments. Histological analysis further revealed layer-specific and group-dependent retinal effects, whereas no significant difference in lens thickness was detected. Cr(VI)-only exposure was associated with retinal thinning: Cr-H showed a thinner total retina than Mix-H, and Cr-L showed reduced IPL and INL thickness relative to Mix-H. In contrast, Mix-H uniquely showed significant ONL thickening relative to VC and differed from several single-exposure groups. Therefore, co-exposure did not simply aggravate Cr(VI)-associated retinal thinning. Instead, the mixture appeared to redirect retinal structural responses, particularly at the high dose.

Interpretation of ocular toxicity also benefits from distinguishing rare severe defects from more common sublethal alterations. In the present study, anophthalmia was observed only once in the Cr-L group (Figure 1D), and was absent in the Cr-H and mixture groups. Given the rarity of this phenotype, this observation should be interpreted cautiously. Nevertheless, it is notable that *rx1* was significantly downregulated only in the Cr-L group relative to VC, whereas no significant *rx1* change was detected in the corresponding mixture groups. This single-exposure-specific response suggests that low-dose Cr(VI) may affect part of the early eye-field developmental program, while co-exposure did not reproduce this molecular pattern. This divergence is biologically plausible in light of the established role of the Rx family in optic vesicle morphogenesis. In teleosts, *rx3* mutants fail to form eyes because optic vesicle development is impaired, and reduced *rx1* and *rx2* expression in *rx3* mutants supports a hierarchical Rx regulatory program during eye morphogenesis [31]. In zebrafish *chokh/rx3* mutants, *rx1* expression is weak and transient and is insufficient to support optic vesicle evagination [32]. In addition, *rx1* has been implicated in retinal progenitor proliferation [31]. Together, these observations support the interpretation that the Cr(VI)-related suppression of *rx1* may be associated with ocular developmental vulnerability under the Cr-L condition. The absence of significant *rx1* downregulation in the mixture groups may further suggest that co-exposure modified, rather than simply intensified, the Cr(VI)-associated molecular response.

The transcriptional results provide preliminary molecular context for the morphological and histological observations, although their explanatory strength is constrained by the sensitivity of the qPCR analysis. As noted in Section 3.4, the qPCR endpoints were powered to detect only very large omnibus effects (*f* ≈ 0.995; Table S5). Therefore, non-significant results for several genes, including *pkcα*, *rho*, *mipa*, *atoh7*, *crybb*, and *aldh1a2*, cannot be taken as evidence that these genes were unaffected by exposure. Rather, they only indicate that no statistically reliable changes were detected under the current qPCR design; moderate or subtle transcriptional responses cannot be excluded.

Within this limitation, the significant transcriptional responses help distinguish endpoint-specific differences between single and combined exposures. Low-dose Cr(VI) significantly induced *cyp26a1* and *rdh1* relative to VC, while Mix-L significantly increased *rdh1* expression. At 100 μg/L, *rdh1* expression in the FB-H and Cr-H groups exceeded that in Mix-H, and Cr-L and Mix-L also showed higher *rdh1* expression than Mix-H. This pattern suggests that the high-dose mixture attenuated selected single-compound transcriptional responses rather than enhancing them. Because RA signaling is essential for ocular and retinal patterning [33], and disturbed RA gradients are associated with abnormal retinal differentiation and architecture [21,34], these findings suggest a possible involvement of RA-related signaling in the retinal alterations observed here.

Lens- and phototransduction-related genes also showed gene-specific responses. *cryaa* was significantly downregulated only in the Mix-L group relative to VC, indicating a mixture-associated response at the low dose. In contrast, *crygn2* was significantly reduced in Cr-H relative to both VC and Mix-L, suggesting a Cr(VI)-dominant lens-related response at the high dose. The downregulation of *crygn2* may be consistent with altered lens structural protein homeostasis [35]. For phototransduction-related genes, *opn1sw* was reduced in FB-L and Cr-L, *opn1lw* was reduced in Mix-L and Cr-H, and *gnat1* differed between FB-H and both mixture groups. These results indicate that F-53B, Cr(VI), and the mixture affected overlapping but non-identical visual-system-related transcriptional endpoints.

The behavioral results provide a functional comparison between single exposure and co-exposure. Locomotor inhibition was mainly driven by Cr(VI), and the mixture did not exceed the impairment caused by Cr(VI) alone. Specifically, reduced swimming speed relative to VC was observed in Cr-L during the dark phase and in Cr-L and Cr-H during the second light phase, whereas the mixture groups did not show significant locomotor inhibition relative to VC. This pattern is consistent with reports that metal exposure disrupts light–dark locomotor activity [23]. Cr(VI) has also been linked to reduced spontaneous swimming together with the inhibition of AChE activity, suggesting impaired neuromuscular transmission [36]. In parallel, changes in cone- and phototransduction-related genes were observed: *opn1sw* was reduced in FB-L and Cr-L, *opn1lw* was reduced in Mix-L and Cr-H, and *gnat1* differed significantly between FB-H and both mixture groups. Taken together, these findings indicate that altered light-responsive behavior in exposed larvae may reflect both neural and visual-system disruption. Importantly, however, the behavioral endpoint remained primarily Cr(VI)-driven, despite mixture-specific changes in retinal structure and gene expression. This again shows that co-exposure did not produce uniform potentiation, but instead generated endpoint-specific divergence across biological levels.

Several limitations should be considered when interpreting these single-exposure and co-exposure comparisons. First, the exposure window was intentionally restricted to early development up to 5 dpf. This window is appropriate for detecting acute developmental, early ocular, behavioral, and transcriptional responses in zebrafish embryos and larvae, as demonstrated by the significant effects observed in several endpoints. However, this design does not address delayed juvenile, adult, or life-cycle outcomes. Therefore, longer-term studies are needed to determine whether the early effects observed here persist, recover, or progress during later development [37]. Second, although the matched-concentration design allowed for direct comparisons between single and combined exposures, pharmacokinetic or toxicokinetic data were not obtained, and internal dosimetry was not measured. Therefore, the present study cannot determine whether co-exposure altered the absorption, distribution, metabolism, or elimination of F-53B or Cr(VI), nor can it rule out the possibility that interactions between the two compounds affected their toxicokinetic behavior and internal exposure levels. The reduced or divergent responses observed for selected mixture endpoints are therefore described as endpoint-dependent toxicological patterns rather than being attributed to a specific absorption-, distribution-, metabolism-, or elimination-related mechanism. Third, behavioral assessment was limited to locomotor responses in a light–dark paradigm. Inclusion of vision-specific functional tests would strengthen the causal link between retinal structure, phototransduction-related gene expression, and visual performance. Fourth, the gene expression analysis should be considered preliminary because the qPCR endpoints were based on three biological replicates per group. Although this design is commonly used for exploratory transcriptional assessment in zebrafish developmental toxicity studies, the G*Power-based sensitivity analysis indicated that only very large effects could be detected with adequate power for these endpoints. Future studies should therefore validate these transcriptional findings using larger sample sizes, time-resolved sampling, and complementary approaches such as protein-level analysis, spatial retinal markers, or transcriptomic profiling. Finally, the present study used two matched concentration levels selected to represent environmentally relevant exposure scenarios and was not designed to establish complete concentration–response surfaces or formal mixture-toxicity models. A broader concentration matrix, including lower, intermediate, and higher concentrations as well as equitoxic mixture designs, would be required to distinguish concentration addition, independent action, and true synergistic or antagonistic interactions more rigorously.

Future studies should extend exposure duration using juvenile, multi-week, or life-cycle designs to determine whether the early mixture effects observed here persist, intensify, or transition into chronic outcomes and adaptive remodeling. Particular attention should be given to molecular targets highlighted in the present study, including *rx1*, especially under Cr-L exposure, other members of the Rx family such as *rx2* and *rx3*, RA-related genes such as *cyp26a1* and *rdh1*, and phototransduction markers such as *opn1sw, opn1lw,* and *gnat1*. Integrating transcript-level analysis with spatially resolved retinal endpoints, including layer-specific cell composition, apoptosis or proliferation indices, and photoreceptor integrity, would provide a more complete view of the mechanisms involved. In addition, vision-related functional assays, such as optokinetic or optomotor responses, contrast sensitivity, and electrophysiological measurements where feasible, would help clarify structure–function relationships under single and mixed exposure conditions. Importantly, biomonitoring data indicate that non-occupational human internal exposure to F-53B and chromium generally occurs at trace to low-μg/L range, but concentrations in highly exposed populations, especially in breast milk from contaminated settings, could reach tens of μg/L and occasionally approach or exceed 100 μg/L [38,39,40,41]. Thus, the nominal concentrations used here, 10 and 100 μg/L, encompass the upper range of environmentally relevant burdens in certain matrices and hotspot scenarios.

## 5. Conclusions

This study demonstrates that F-53B, Cr(VI), and their mixtures disrupt zebrafish early development across multiple biological levels, but the effects of co-exposure cannot be predicted by simply summing or ranking the effects of the two single compounds. Single Cr(VI) exposure mainly drove hatching suppression, locomotor inhibition, retinal-layer thinning, and several ocular-development-related transcriptional responses. F-53B alone produced measurable developmental and ocular effects, but these responses were generally more selective. In contrast, F-53B/Cr(VI) co-exposure was characterized by enhanced malformation, increased heart rate, and specific ocular and retinal alterations, while some Cr(VI)-dominant responses, including locomotor impairment and selected transcriptional changes, were not further aggravated by the mixture. These findings indicate that co-exposure produced an endpoint-dependent toxicity profile, with both enhanced and attenuated responses. The Cr-L-specific downregulation of *rx1* and the rare occurrence of anophthalmia provide a preliminary clue that low-dose Cr(VI) may interfere with early eye-field-related developmental programs. Further studies using broader concentration matrices, gene expression analysis at multiple developmental time points, spatial retinal analyses, internal dosimetry, and vision-specific functional assays are needed to clarify the mechanisms underlying these mixture-specific responses. Overall, combined contaminant toxicity should be evaluated within an endpoint- and pathway-dependent framework.

## Figures and Tables

**Table 1:** Target genes analyzed by qPCR in this study.

Gene Symbol	Full Name	Functional Category
*rdh1*	Retinol dehydrogenase 1	Retinoic acid metabolism
*aldh1a2*	Aldehyde dehydrogenase 1 family member a2	Retinoic acid synthesis
*cyp26a1*	Cytochrome P450 family 26 subfamily a member 1	Retinoic acid catabolism
*cryaa*	Crystallin alpha A	Lens development/lens structural protein
*crybb*	Crystallin beta B	Lens development/lens structural protein
*crygn2*	Crystallin gamma N2	Lens development/lens structural protein
*mipa*	Major intrinsic protein of lens fiber a	Lens fiber differentiation
*pax6*	Paired box 6a	Eye development
*pkcα*	Protein kinase C alpha	Retinal signaling/phototransduction
*gnat1*	Guanine nucleotide-binding protein alpha transducing activity polypeptide 1	Phototransduction
*rx1*	Retinal homeobox 1	Eye-field specification/retinal progenitor development
*rho*	Rhodopsin	Rod photoreceptor marker
*opn1sw*	Opsin 1 short-wave-sensitive 1	Cone photoreceptor marker
*opn1lw*	Opsin 1 long-wave-sensitive 1	Cone photoreceptor marker
*atoh7*	Atonal bHLH transcription factor 7	Retinal ganglion cell differentiation
*β-actin*	Beta-actin	Reference gene

**Table 2:** Developmental and ocular morphometric endpoints after exposure to F-53B and Cr(VI) from 0 to 120 hpf in zebrafish. Data represent the mean ± SD of three independent replicates. Statistical significance is indicated as follows: * *p* < 0.05, ** *p* < 0.01, *** *p* < 0.001 compared with VC; ^aa^ *p* < 0.01 compared with Mix-L; and ^b^ *p* < 0.05, ^bb^ *p* < 0.01 and ^bbb^ *p* < 0.001 compared with Mix-H.

	Time	VC	FB-L	Cr-L	Mix-L	FB-H	Cr-H	Mix-H
Hatching rate (%)	72 hpf	94 ± 3	77 ± 12 *	71 ± 14 **	75 ± 4 ***	89 ± 8 ^b^	76 ± 15 *	80 ± 10 *
Malformation rate (%)	72 hpf	1.30 ± 0.50	5.80 ± 1.00 **	6.50 ± 1.30 ***	7.50 ± 1.30 ***	9.80 ± 2.10 ***^,bbb^	11.30 ± 1.50 ***^,bb^	14.00 ± 1.80 ***
Cumulative mortality (%)	72 hpf	9 ± 4	21 ± 11 ***	25 ± 8 ***	26 ± 8 ***	24 ± 10 ***	26 ± 11 ***	28 ± 9 ***
Spontaneous coiling (coils/min)	24 hpf	2.66 ± 0.21	2.36 ± 0.25	1.98 ± 0.28 ^aa^	2.76 ± 0.35	2.21 ± 0.28	1.96 ± 0.36 ^aa^	2.32 ± 0.2
Heart rate (beats/min)	72 hpf	226 ± 10	248 ± 11 ^aa^	264 ± 13 **	277 ± 14 ***	258 ± 9 **^,bbb^	265 ± 6 **^,bbb^	310 ± 30 ***
Body length (μm)	120 hpf	4026 ± 165	4151 ± 93	4028 ± 173	4075 ± 137	4155 ± 150	4171 ± 168	4219 ± 62 *
Head width (μm)	120 hpf	415 ± 28	432 ± 22 *	402 ± 25	412 ± 20	417 ± 21 ^b^	433 ± 27 *	440 ± 32 **
Eye diameter X (μm)	120 hpf	324 ± 18	353 ± 14 **	329 ± 31	341 ± 13	351 ± 13 **	354 ± 26 **	350 ± 15 **
Eye diameter Y (μm)	120 hpf	272 ± 14	300 ± 14 *	287 ± 25	287 ± 15	290 ± 16 ^b^	295 ± 25 **	308 ± 22 ***

## Data Availability

The original contributions presented in this study are included in the article and Supplementary Materials. Further inquiries can be directed to the corresponding authors.

## Supplementary Materials

The following supporting information can be downloaded at: https://www.mdpi.com/article/10.3390/toxics14060471/s1, Figure S1. Schematic illustration of ocular morphometric measurements and representative retinal histological structure in zebrafish larvae. (A) Measurement of horizontal and vertical eye diameters and head width. (B) Representative retinal histological structure showing the lens and retinal layers, including the ganglion cell layer (GCL), inner plexiform layer (IPL), inner nuclear layer (INL), outer nuclear layer (ONL); Table S1. Comparison between the untreated control (SRW only) and vehicle control (SRW containing 0.01% DMSO) across major endpoints in zebrafish; Table S2. F-53B and Cr(VI) concentration in exposure solution; Table S3. List of primers used in this study; Text S1. F-53B and Cr(VI) analysis; Text S2. Gene expression assays (provided in Supplementary Materials, SM1, Word format); Table S4. Assumption checks, transformation decisions, omnibus tests, and selected reference post hoc results for seven analyzed groups; Table S5. G*Power-based sensitivity analysis for sample-size justification (provided in Supplementary Materials, SM2, Excel format).
