# Supplementary material for: Combined Developmental Toxicity of Ecologically Relevant Concentrations of the PFOS Alternative F-53B and Hexavalent Chromium in Zebrafish, Danio rerio
Source: Toxics. 2026 May 27;14(6):471. doi: 10.3390/toxics14060471 (PMC13307901; doi:10.3390/toxics14060471)
Supplement: Supplementary file 1 [file toxics-14-00471-s001.zip › SM1 supplementary_edited.pdf]

Combined Developmental Toxicity of Ecologically Relevant Concentrations of the PFOS Alternative F-53B and Hexavalent Chromium in Zebrafish, Danio rerio

Qunjie Feng [1,2,3,†], Ximei Wu [1,†], Ming Chen [1], Hui Li [1], Wei Tong [1], Yanhong Gao [1], Wenli Li [1], Zenghua Qi [2,3], Chaoyang Long [1,*] and Yingxin Yu [1,2,3,*]

[1] Guangdong-Hong Kong-Macao Joint Laboratory for Contaminants Exposure and Health, Guangdong Provincial Center for Disease Control and Prevention, Guangzhou 510430, China
[2] Guangdong-Hong Kong-Macao Joint Laboratory for Contaminants Exposure and Health, Guangdong Key Laboratory of Environmental Catalysis and Health Risk Control, Institute of Environmental Health and Pollution Control, Guangdong University of Technology, Guangzhou 510006, China
[3] Guangdong Basic Research Center of Excellence for Ecological Security and Green Development, Key Laboratory of City Cluster Environmental Safety and Green Development, School of Environmental Science and Engineering, Guangdong University of Technology, Guangzhou 510006, China
* Correspondence: chaoyang_long@163.com (C.L.); yuyingxin@gdut.edu.cn (Y.Y.); Tel.: +86-020-31051238 (C.L.); +86-020-39322295 (Y.Y.)
† These authors contributed to the work equally and should be regarded as co-first authors.

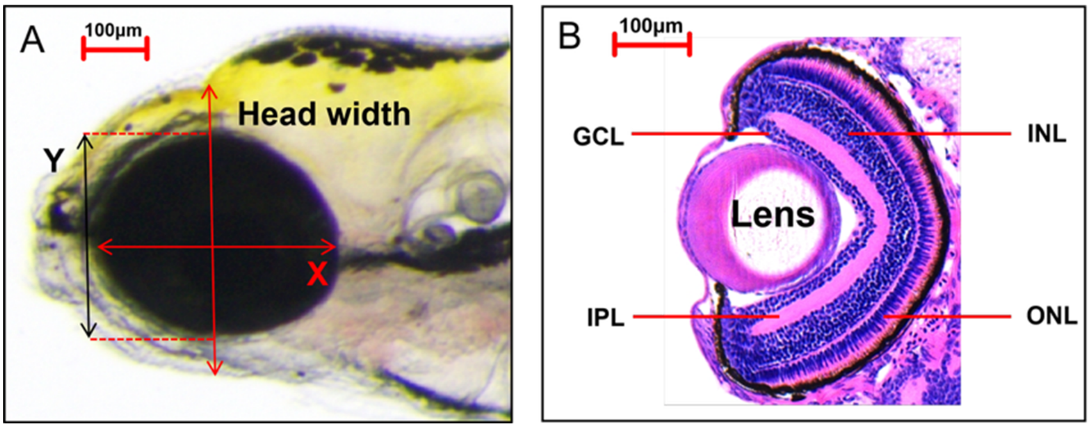

Figure S1. Schematic illustration of ocular morphometric measurements and representative retinal histological structure in zebrafish larvae. (**A**) Measurement of horizontal and vertical eye diameters and head width. (**B**) Representative retinal histological structure showing the lens and retinal layers, including the ganglion cell layer (GCL), inner plexiform layer (IPL), inner nuclear layer (INL), and outer nuclear layer (ONL).

Table S1. F-53B and Cr(VI) concentrations in exposure solution.

Exposure group	Nominal concentration (μg/L)	Measured F-53B concentration (μg/L)	Measured Cr(VI) concentration (μg/L)
10 μg/L F-53B	10.00	9.31 ± 0.98	N.D.
10 μg/L Cr(VI)	10.00	N.D.	9.35 ± 0.45
10 μg/L Mixture	10.00 + 10.00	9.95 ± 0.86	9.41 ± 0.42
100 μg/L F-53B	100.00	110.09 ± 3.17	N.D.
100 μg/L Cr(VI)	100.00	N.D.	96.21 ± 2.59
100 μg/L Mixture	100.00 + 100.00	109.49 ± 4.21	95.46 ± 0.88
Control	0	N.D.	N.D.

N.D. = Not detected

Table S2. List of primers used in this study.

Primers	5`- 3`
Rdh1	F: GGAGGATACTGCCTGTCCAA<br>R: CTCCAGCGTTTCTTCAGGTC
Aldh1a2	F: CGACAAGGCTGATGTGGATAAAG<br>R: TTCCTCCATACTGAACCCAGAGA
Cyp26a1	F: AGTGGCCAGCATCAGTGAGAA<br>R: GAACGCCCTCATAATGGCCT
Cryaa	F: TTGACCAACCTGGTGACAGA<br>R: TTAGGGAGAAAGCGGAGGTT
Crybb	F: GACTAACTTCCGTGGTGAGATG<br>R: GTCTGAGGGACATGAGACAATC
Crygn2	F:GAGTACCCCGAATTCCAGCG<br>R: GCGGAAGTTAGGCTCCTCAT
Mipa	F: GTCCCTGTTTCGTGCCTTCT<br>R: GGCTGAAGCGTATTCAAGGC
Pax6	F: CAACACGGTTAACGTCCGAG<br>R: CCAGTCCAACAAGGGAACCT
Rx1	F: CCTCCCTTCACCGACAAATAC<br>R: TGGATATGCTCCTTGGCTTTC
Gnat1	F: GCGAGTGGTCATCCAAAAGG<br>R: TTGTGCTTTTCCCCGACTCA

Rho	F: CCTGCCTCGTTTCTTCACA<br>R: TGGGTATTCGTATGGGCTC
Opn1sw	F: CTTTGCTATGCCCCTTATGC<br>R: ATGAGGTTTCGGTCTTGCTG
Opn1lw	F: TAGCACTGCTTGAGGGAATGT<br>R: GGGCAGGCATCTACCTATCAC
Pkcα	F: GGACGCAGAGGGTCATATTAAG<br>R: GTAGTCTGGCGTTCCACAAA
Atoh7	F: TTGAAGAGCCATGAAGCCCC<br>R: ACTTCTCCGGGTCTCTGGAA
β-Actin	F: AGGTCATCACCATTGGCAAT<br>R: GATGTCGACGTCACACTTCAT

Table S3. Comparison between the untreated control (SRW medium only) and vehicle control (SRW containing 0.01% DMSO) across major endpoints in zebrafish.

Endpoint	Untreated control (mean ± SD)	n	Vehicle control (mean ± SD)	n	Selected test	Test statistic	p value	Effect size	Significant at p < 0.05
Hatching rate (%)	94.20 ± 4.81	12	94.11 ± 3.22	12	Mann–Whitney U test	68	0.8387	rank-biserial r = −0.056	No
Malformation rate (%)	1.75 ± 0.50	4	1.25 ± 0.50	4	Mann–Whitney U test	12	0.2471	rank-biserial r = 0.500	No
Cumulative mortality (%)	7.56 ± 5.07	18	9.12 ± 3.66	18	Student's t-test	−1.06	0.2965	Hedges' g = −0.346	No
Spontaneous coiling (coils/min)	2.653 ± 0.174	6	2.658 ± 0.211	6	Student's t-test	−0.029	0.9779	Hedges' g = −0.019	No
Heart rate (beats/min)	230.17 ± 7.96	6	226.00 ± 9.59	6	Student's t-test	0.819	0.432	Hedges' g = 0.436	No
Body length (μm)	4129 ± 139	11	4026 ± 165	11	Student's t-test	1.541	0.1398	Hedges' g = 0.646	No
Head width (μm)	395 ± 19	11	415 ± 28	11	Mann–Whitney U test	80	0.0844	rank-biserial r = 0.455	No

Eye diameter X (μm)	319 ± 16	11	324 ± 18	11	Student's t-test	0.77	0.4505	Hedges' g = 0.323	No
Eye diameter Y (μm)	271 ± 9	11	272 ± 14	11	Student's t-test	0.237	0.8151	Hedges' g = 0.099	No
Lens thickness (μm)	241 ± 23	14	246 ± 19	14	Welch's t-test	−0.379	0.7104	Hedges' g = −0.152	No
Total retinal thickness (μm)	145 ± 12	14	138 ± 10	14	Mann–Whitney U test	117	0.0946	rank-biserial r = 0.393	No
GCL thickness (μm)	31.433 ± 7.393	14	28.079 ± 4.237	14	Student's t-test	1.446	0.1611	Hedges' g = 0.551	No
IPL thickness (μm)	35.883 ± 5.584	14	32.743 ± 3.919	14	Student's t-test	1.679	0.1061	Hedges' g = 0.640	No
INL thickness (μm)	57.342 ± 8.757	14	53.257 ± 5.787	14	Mann–Whitney U test	108	0.2268	rank-biserial r = 0.286	No
ONL thickness (μm)	24.925 ± 2.827	14	23.379 ± 1.777	14	Student's t-test	1.696	0.1029	Hedges' g = 0.646	No
Swimming speed, $Light_1$ (mm/s)	2.665 ± 0.848	12	2.745 ± 0.783	12	Student's t-test	−0.222	0.8271	Hedges' g = −0.095	No
Swimming speed, Dark (mm/s)	5.102 ± 1.177	12	4.959 ± 0.636	12	Student's t-test	0.37	0.7148	Hedges' g = 0.146	No

Swimming speed, Light$_2$ (mm/s)	2.395 ± 0.881	12	2.165 ± 0.615	12	Student's t-test	0.742	0.4662	Hedges' g = 0.292	No
CYP26A1	0.396 ± 0.049	3	0.372 ± 0.125	3	Student's t-test	0.309	0.7728	Hedges' g = 0.202	No
P53	1.725 ± 0.451	3	1.853 ± 0.653	3	Student's t-test	−0.279	0.7938	Hedges' g = −0.182	No
CASP3	2.523 ± 0.490	3	2.956 ± 0.224	3	Student's t-test	−1.394	0.2358	Hedges' g = −0.910	No
CASP9	1.497 ± 0.347	3	1.393 ± 0.373	3	Student's t-test	0.356	0.7397	Hedges' g = 0.233	No
RDH1	2.353 ± 0.251	3	2.539 ± 0.578	3	Mann–Whitney U test	3	0.7	rank-biserial r = −0.333	No
ALDH1A2	1.337 ± 0.377	3	1.277 ± 0.182	3	Student's t-test	0.249	0.8154	Hedges' g = 0.163	No
MIPA	3.962 ± 0.775	3	3.931 ± 1.236	3	Student's t-test	0.038	0.9717	Hedges' g = 0.025	No
CRYGN2	4.921 ± 0.583	3	5.257 ± 1.103	3	Student's t-test	−0.466	0.6655	Hedges' g = −0.304	No
CRYBB	0.878 ± 0.068	3	0.926 ± 0.177	3	Student's t-test	−0.444	0.68	Hedges' g = −0.290	No

CRYAA	2.020 ± 0.313	3	2.104 ± 0.454	3	Student's t-test	−0.265	0.8043	Hedges' g = −0.173	No
PAX6	0.948 ± 0.117	3	0.930 ± 0.128	3	Student's t-test	0.175	0.8695	Hedges' g = 0.114	No
RX1	1.656 ± 0.263	3	1.739 ± 0.155	3	Mann–Whitney U test	4	1	rank-biserial r = −0.111	No
OPN1SW	3.201 ± 0.685	3	2.759 ± 0.484	3	Student's t-test	0.912	0.4133	Hedges' g = 0.596	No
OPN1LW	2.939 ± 0.738	3	2.490 ± 0.238	3	Mann–Whitney U test	7	0.4	rank-biserial r = 0.556	No
PKCA	1.341 ± 0.428	3	1.389 ± 0.169	3	Student's t-test	−0.182	0.8643	Hedges' g = −0.119	No
ATOH7	2.994 ± 0.987	3	2.448 ± 0.448	3	Student's t-test	0.872	0.4325	Hedges' g = 0.570	No
GNAT1	4.897 ± 0.355	3	5.433 ± 0.802	3	Student's t-test	−1.059	0.3494	Hedges' g = −0.692	No
RHO	2.604 ± 0.247	3	2.471 ± 0.410	3	Mann–Whitney U test	7	0.4	rank-biserial r = 0.556	No

Data are presented as mean ± SD. Untreated control: SRW only; vehicle control: SRW containing 0.01% DMSO. Normality and homogeneity of variance were assessed using the Shapiro–Wilk test and Levene's test, respectively. Student's t-test was used for normally distributed data with homogeneous variances, Welch's t-test for normally distributed data with unequal variances, and Mann–Whitney U test for non-normally distributed data. Effect sizes are reported as Hedges' g for parametric comparisons and rank-biserial r for nonparametric comparisons.

Text S1. F-53B and Cr(VI) analysis.

1) F-53B analysis

Exposure media were sampled immediately before daily renewal, mixed thoroughly, and diluted with ultrapure water when necessary. For F-53B determination, aliquots were fortified with a fixed volume of MPFAC-MXA (a mixture of 19 isotope-labelled PFAS internal standards; Wellington Laboratories, Canada) to correct for matrix effects and instrumental drift. After vortexing and brief centrifugation, 100 μL of the supernatant was transferred into polypropylene autosampler vials for UHPLC-MS/MS analysis.

F-53B was quantified by ultra-high-performance liquid chromatography coupled with tandem mass spectrometry (UHPLC-MS/MS). An Agilent 1290 UHPLC system (Agilent Technologies, Santa Clara, CA, USA) equipped with a Zorbax Eclipse Plus C18 column (1.8 μm, 50 mm × 2.1 mm) and an Ascentis Express F5 PFP guard column (2.7 μm, 30 mm × 2.1 mm; Sigma-Aldrich) were used. Both analytical and guard columns were maintained at 30 °C. To reduce background PFAS originating from the LC system, an additional Zorbax Eclipse Plus C18 column (5 μm, 50 mm × 4.6 mm) was installed in series upstream of the injector and used as a delay/trap column. The mobile phase consisted of 20 mM ammonium formate in water (solvent A) and methanol (solvent B). The flow rate was 0.30 mL/min. The gradient program started at 55% A/45% B, held briefly, then increased linearly to 60% B at 3 min and to 63% B at 15 min. The proportion of B was then ramped to 95% at 15.5 min and held until 18.5 min to elute strongly retained compounds. The system was returned to initial conditions

at 19 min and re-equilibrated for 4 min before the next injection. The injection volume was 5 μL.

Detection was performed on an Agilent 6495B triple-quadrupole MS (Agilent Technologies, Palo Alto, CA, USA) equipped with an electrospray ionization source operated in negative ion mode. F-53B was monitored in multiple reaction monitoring (MRM) mode using optimized precursor/product ion transitions and compound-specific collision energies.

2) Cr(VI) analysis

Dissolved Cr(VI) in exposure solutions was measured by ion chromatography coupled with post-column derivatization and photometric detection. Exposure media were collected before renewal, mixed, and diluted with ultrapure water to fall within the linear calibration range. Samples were analyzed as soon as possible after sampling.

Chromatographic separation of Cr(VI) was achieved on a dedicated anion-exchange column for hexavalent chromium (hexavalent chromium analytical column, model ELSpe-2) installed on an ultra-trace Cr(VI) analyzer. The eluent was an ammonium nitrate solution (100 mmol/L) delivered at a flow rate of 1.2 mL/min. After column separation, a post-column derivatization stream containing 1,5-diphenylcarbazide (0.3–0.4 g dissolved in 50 mL methanol, diluted with 450 mL ultrapure water and acidified with 6 mL concentrated sulfuric acid) was introduced at 0.7 mL/min. Under strongly acidic conditions, Cr(VI) is reduced and forms a purple Cr(III)–diphenylcarbazone complex, which was detected spectrophotometrically at 540 nm in a flow cell.

For each sample, an injection volume of 900 μL was used, and the total analysis time was approximately 220 s per run. Quantification was performed using external calibration with a series of potassium dichromate standards prepared in the same matrix as the samples. Calibration curves were checked for linearity before and after each sample batch. Method blanks and spiked samples were analyzed in parallel to evaluate potential contamination and matrix recovery.

Text S2. Gene expression assays.

For gene expression analysis, larvae were sampled at 120 hpf. Within each treatment group, approximately 50 larvae were randomly collected and pooled to constitute one biological sample. Two pools were prepared per replicate, yielding a total of six pooled samples per treatment group. Larvae were briefly rinsed in SRW, blotted to remove excess medium, snap-frozen in liquid nitrogen, and stored at −80 °C until RNA extraction.

Total RNA was isolated using TRIzol reagent (Invitrogen, Carlsbad, CA, USA) following the manufacturer's instructions. The RNA pellets were resuspended in nuclease-free water and treated with RNase-free DNase I (Thermo Scientific, USA) to remove residual genomic DNA. RNA concentration and purity were assessed using a NanoDrop 2000 spectrophotometer (Thermo Fisher Scientific, USA), and only samples with an A260/A280 ratio between 1.9 and 2.1 were used for downstream analysis.

For each sample, 500 ng of total RNA was reverse-transcribed into first-strand cDNA using a commercial reverse transcription kit suitable for qPCR (ReverTra Ace® qPCR RT kit, TOYOBO, Japan), following the manufacturer's protocol. Gene-specific primers are listed in Supplementary Table S1. Quantitative real-time PCR was performed with a SYBR® Green-based master mix (SYBR® Green Realtime PCR Master Mix, TOYOBO, Japan) on a QuantStudio 7 Flex Real-Time PCR System (Thermo Fisher Scientific, USA).

Each qPCR reaction was run in duplicate or triplicate in a final volume of 10–20 μL. The thermal cycling protocol consisted of an initial denaturation at 95 °C for 1 min,

followed by 40 cycles of 95 °C for 15 s and 60 °C for 60 s. At the end of amplification, a melt-curve analysis was performed to confirm the specificity of each amplicon. β-actin was used as the reference (housekeeping) gene. For each target gene, cycle threshold (C_t) values were normalized to β-actin (ΔC_t), and relative expression levels were calculated using the $2^{-\Delta\Delta Ct}$ method with the solvent control group as calibrator. Only reactions with a single melt-curve peak and consistent technical replicates were included in the final analysis.
